# Draft-genome sequence of *Bacillus subtilis* SS17, a promising shrimp probiotic candidate

**DOI:** 10.1128/mra.00031-25

**Published:** 2025-06-24

**Authors:** Md Mahbubur Rahman, Ms Khadiza Sarkar, Sulav Indra Paul, Umma Habiba Tanjum, Bishwajit Karmakar Sunny, Soharth Hasnat, Mohammad Abdus Salam, Tofazzal Islam, Md Zia Haider Chowdhury, Mohammed Shariful Azam, Taslima Akter, Md Mohidul Islam, Md Abdul Hannan

**Affiliations:** 1 Institute of Biotechnology and Genetic Engineering, Gazipur Agricultural University 198780, Gazipur, Bangladesh; 2 Department of Genetics and Fish Breeding, Gazipur Agricultural University 198780, Gazipur, Bangladesh; 3 Department of Fisheries, Government of the People's Republic of Bangladesh, Matshya Bhaban, Dhaka, Bangladesh; 4 Department of Aquatic Animal Health Management, Sher-e-Bangla Agricultural University 54494, Dhaka, Bangladesh; University of Maryland School of Medicine, Baltimore, Maryland, USA

**Keywords:** *Bacillus subtilis*, Shrimp probiotic, *Penaeus monodon*, Shrimp gut

## Abstract

We describe a promising shrimp probiotic candidate *Bacillus subtilis* SS17, which was isolated from the intestine of a healthy tiger shrimp, *Penaeus monodon*. The *de novo* assembly resulted in an estimated chromosome size of 4,042,212 bases and 4,215 CDS. The genome sequence information will help exploit the probiotic potential of the strain.

## ANNOUNCEMENT

Probiotics have been reported to promote growth, immunity, and disease resistance in shrimp (1, 2) and are considered one of the effective and eco-friendly approaches for controlling bacterial and viral pathogens (3). Most common shrimp probiotics are isolated from the intestine of shrimp and shrimp culture systems (4). SS17 was isolated from the intestine of a healthy *Penaeus monodon* from a shrimp farm at Shamnagar, Satkhira, Bangladesh.

The shrimp intestine was cut aseptically, and 1 g homogenate of the intestine was serially diluted and inoculated on de Man, Rogosa and Sharpe (MRS) agar plate and incubated at 28°C for 48 h to isolate SS17. A single colony was picked from the growing colonies on the MRS plate. The isolate was able to hydrolyze starch, casein, and bile salt in *in vitro* assays. In a feeding experiment in farmers’ fields, higher production was obtained in shrimp when SS17 and another *Bacillus* sp. strain SS45 were applied together with commercial feed compared with the control (5).

To isolate high-quality genomic DNA, a single colony of SS17 was inoculated in nutrient broth and incubated at 28°C for 48 h. The genomic DNA of SS17 was extracted using a GeneJET genomic DNA purification kit (Thermo Fisher Scientific, USA) according to the manufacturer’s instructions. The quality and quantity of the DNA were determined using a NanoDrop spectrophotometer (Thermo Fisher Scientific). A paired-end DNA library was prepared using the Nextera XT Library Prep Kit (Illumina, San Diego, CA, USA) protocol (6). Genome sequencing (600 cycles) was performed using the Illumina MiSeq benchtop sequencer, yielding a total of 254,629 spots with 113.3M bases. Default parameters were used for all bioinformatic software unless otherwise noted. FastQC V.12.0 (7) and Trimmomatic v.0.38 (8) were used to determine the quality of the raw reads and trim the low-quality sequences. Genome assembly was performed using SPAdes v.3.9.0 with multiple k-mer sizes (21, 33, 55, and 77) and the careful mode to reduce mismatches and short indels (9). Quality of the assembled genome was checked by using QUAST v.5.0.2 (10). The NCBI Prokaryotic Genome Annotation Pipeline (PGAP) (https://www.ncbi.nlm.nih.gov/genome/annotation_prok/) was used to annotate the genome (11).

The *de novo* assembly of strain SS17 represented an estimated genome size of 4,042,212 bp with 43.7% G + C content, 57 contigs (largest contig: 714,249 bp and smallest contig: 255 bp), and 281 × genome coverage. The N_50_ and L_50_ values of the assembly were 490,687 and 4, respectively. A total of 4,251 genes including 4,131 total CDSs (4,031 coding genes and 100 pseudogenes), and 120 RNA genes (82 tRNAs, 33 rRNAs, and five noncoding RNAs) were identified in the genome. RAST v.2.0 (12) projected 325 subsystems and 4,215 protein-coding genes. ANI analysis using OrthoANIu showed 98% similarity between the query genome and *Bacillus subtilis* (BioSample ID: SAMEA3138188), confirming the strain’s taxonomic identity and supporting its genomic characterization. However, the SS17 genome codes for several orthologs to intrinsic genes of antimicrobial peptides, including surfactin, bacilysin, bacillibactin, subtilosin A, fengycin, and pulcherriminic acid by antiSMASH v6.0 (13). BAGEL4 (14) identified sactipeptides, sporulation-killing factor (*skfA*), comX4, and subtilosin (*SboX*) in the genome. CRISPRCasFinder (15) identified two CRISPR sequences in SS17. No plasmids were identified in the genome by using PlasmidFinder v2.0 (16). VFDB (17) and ResFinder v4.1 (18) did not find any genes related to immune evasion or antibiotic resistance in the genome. These genetic characteristics of the strain suggest its potential for safe use as a probiotic in shrimp aquaculture.

## Data Availability

The whole-genome shotgun project of *Bacillus subtilis* SS17 has been deposited at GenBank under the accession number JBJGEA000000000. The associated BioProject and BioSample accession numbers are PRJNA1185302 and SAMN44701562, respectively. The raw data are available from the Sequence Read Archive (SRA) under accession number SRX26745871.
